# A Review on Ultra-High-Strength Aluminum Alloys for Aerospace Applications: Forming, Microstructure, and Mechanical Properties

**DOI:** 10.3390/ma19091809

**Published:** 2026-04-29

**Authors:** Xuanxi Xu, Huabiao Chen, Linzhi Tang, Li Wang, Xiaoxiao Fu, Hongwei Ran, Daoxiang Wu, Hua Zhou, Guoqiang You

**Affiliations:** 1 Chinalco Southwest Aluminium (Group) Co., Ltd., Chongqing 401326, China; 13308376450@163.com (X.X.);; 2 National Engineering Research Center for Magnesium Alloys, College of Materials Science and Engineering, Chongqing University, Chongqing 400044, China; 3 National Key Laboratory of Advanced Casting Technologies, Chongqing University, Chongqing 400044, China

**Keywords:** aerospace, ultra-high-strength aluminum alloys, mechanical property, precipitation, heat treatment

## Abstract

The increasing demand for lightweight aerospace structures has driven the continuous development of ultra-high-strength aluminum alloys (UHSAAs). Owing to their low density and high specific strength, UHSAAs remain the primary materials for next-generation aerospace structural components. Over the past decades, their tensile strength has increased from the 500 MPa level to beyond 700 MPa, accompanied by a shift in research focus from strength maximization to the synergistic optimization of strength, ductility, and damage tolerance. This work concentrates on 7xxx series and Al–Li alloys, systematically reviewing recent research advances and key challenges in alloy design and forming. Particular emphasis is placed on new strategies for strength–ductility synergy and the associated microstructural strengthening and toughening mechanisms. Finally, future development directions are discussed to provide guidance for the design and engineering application of high-performance aerospace aluminum alloys.

## 1. Introduction

As a strategic emerging industry, the development level of the aerospace manufacturing industry is an important indicator of a nation’s overall technological and industrial strength [1,2,3,4]. Owing to their high specific strength, low density, excellent mechanical properties, and good formability, aluminum alloys represent a class of mature lightweight high-performance materials that have been extensively used in aerospace applications [5]. With increasingly stringent requirements for comprehensive service performance and environmental adaptability of aerospace vehicles, UHSAAs are being continuously advanced toward higher performance, larger-scale applications, and further weight reduction [6,7].

UHSAA were developed in the 1960s to meet the requirements of aerospace applications. Aluminum alloys with yield strengths (YSs) above 550 MPa are generally classified as UHSAAs [8,9]. At present, industrial UHSAAs are dominated by the 7xxx series, namely Al–Zn–Mg–Cu-based alloys strengthened by the precipitation of η′/η (MgZn_2_) phases. Owing to their high strength-to-weight ratio, these alloys are widely used in critical aerospace structural components, such as aircraft wings, fuselage frames, and landing gear, which enables them to substitute for more expensive titanium alloys in many structural applications [10]. During the 1980s, escalating fuel costs necessitated further reductions in structural weight, while elevated operational speeds imposed higher service-temperature demands on aluminum alloys. Under these conditions, aluminum–lithium (Al–Li) alloys attracted considerable attention owing to their low density and high specific strength, as Li is the lightest metallic element [11,12,13,14]. Compared with conventional Al alloys, Al–Li alloys can reduce component weight by 10–20% while increasing stiffness by 15–20% and are therefore regarded as next-generation lightweight structural materials [15]. Consequently, ultra-high-strength 7xxx series alloys (e.g., 7075, 7050, 7085, and 7091), together with advanced third-generation Al–Li alloys, currently dominate aerospace aluminum applications, accounting for more than half of the total aluminum usage and constituting critical ultra-high-strength structural materials in the aerospace manufacturing industry [16].

One generation of materials enables one generation of equipment. Since the 21st century, high-performance structural materials have continuously improved along with the rapid development of aerospace technology. Breakthroughs in emerging materials have, in turn, accelerated the upgrading of conventional materials, placing traditional Al alloys under increasing pressure. The development of next-generation large commercial aircraft imposes more stringent requirements on the in-service reliability of aluminum alloys, demanding not only high specific strength but also high ductility, damage tolerance, fracture toughness, and damage tolerance [17,18]. However, owing to intrinsic limitations associated with strengthening mechanisms and microstructural characteristics, UHSAAs generally suffer from poor fracture toughness and stress corrosion resistance. Moreover, during practical manufacturing, achieving a synergistic improvement in strength and ductility remains a persistent challenge. For example, in extruded 2050 Al–Li alloy, the introduction of a high density of substructures by extrusion markedly increases strength (YS up to ~650 MPa) but typically at the expense of ductility, with elongation (EL) usually below 5% [15,19,20,21]. Evidently, conventional processing routes are no longer sufficient to meet the increasingly demanding requirements of aerospace applications. Developing new alloy systems and advanced preparation processes is an effective way to achieve the strength and ductility synergy in UHSAAs. Consequently, optimizing alloy composition, promoting multi-scale structure, and developing advanced forming technologies and innovative thermomechanical treatments have become key directions in the advancement of high-performance aluminum alloys.

Although UHSAAs for aerospace applications have been reviewed previously (e.g., the perspectives of Dursun et al. and Zhang et al. [1,2]), the new methods for achieving a strength–ductility synergy have not been in detail discussed. Moreover, most existing reviews on UHSAAs were published more than five years ago and lack summaries of recent research progress. Therefore, the objective of this review is to summarize recent advances in ultra-high-strength aluminum alloys for aerospace applications, with particular emphasis on 7xxx series alloys and Al–Li alloys. The relationships among alloy design, forming, microstructural evolution, and mechanical properties are discussed to provide guidance for the development of next-generation high-performance aerospace aluminum alloys.

## 2. Research and Development of High-Strength Aluminum Alloys

UHSAAs are generally defined as Al alloys with YS exceeding 550 MPa, designed primarily through alloying with Cu, Zn, Mg, and Li. They mainly comprise the 7xxx series and Al–Li alloys and were developed to meet the aerospace demands for weight reduction and high load-bearing capacity. Owing to their superior mechanical performance, UHSAAs are widely used in critical load-bearing aerospace structures [22,23].

**Figure 1:**
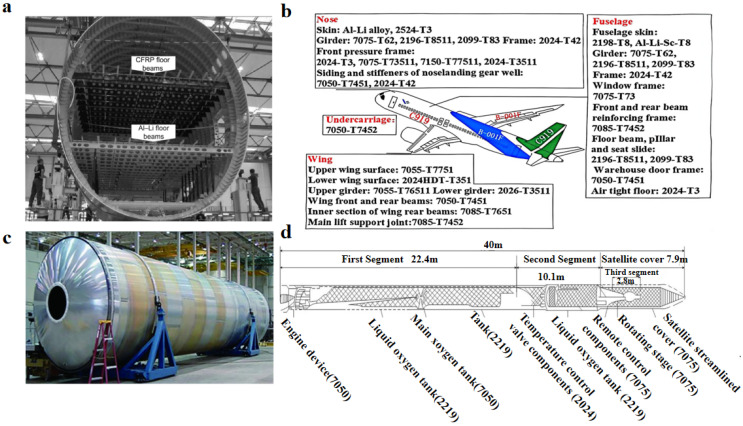
Applications of UHSAAs in aerospace industry: (**a**) floor beams; (**b**) C919 aircraft; (**c**) Rocket fuel tank; (**d**) H-1 rocket [3,29,30,31].

In the aviation sector, Al alloys remain the dominant structural materials, typically accounting for 60–80% of the total aircraft mass. For example, the Al alloy contents in the Boeing 747, Airbus A320, and China’s C919 are approximately 81, 77, and 65 wt%, respectively [24,25]. The application proportions of different Al alloy series in the C919 aircraft is shown, where 2xxx, 7xxx, and Al–Li alloys constitute the primary material systems. Owing to the distinct load requirements of structural components, Al alloy selection exhibits clear functional partitioning, with UHSAAs accounting for approximately 53% of total aluminum usage [3,24]. As shown in Figure 1b, high-load components such as landing gear, fuselage beams, and frame forgings, which are primarily subjected to tensile and bending stresses, predominantly employ 7xxx series alloys, including 7050-T7452, 7075-T62, and 7150-T77511 [26]. In contrast, Al–Li alloys are extensively applied in forward fuselage structures, skin panels, and floor beams (Figure 1a), with representative alloys including 2195-T8, 2099-T83, and Al–Li–Sc-T8 [27,28].

In the space sector, the continuous pursuit of higher payload efficiency and lower launch costs has further intensified the demand for structural weight reduction, which can be directly translated into increased payload capacity. Consequently, the application of Al–Li alloys has expanded in propellant tanks, cylindrical shells, and large thin-walled structures. Figure 1c shows fuel tank structures fabricated from 2195 Al–Li alloy in modern rockets such as SpaceX Falcon 9. Meanwhile, Figure 1d illustrates Al alloy applications in Japan’s H-1 rocket, where 7xxx series alloys remain widely used in load-bearing components. Notably, 7xxx series UHSAAs are still indispensable for critical high-load joints; however, owing to their relatively high density and limited weight-saving potential, their overall proportion in rocket structures tends to gradually decrease.

### 2.1. 7xxx Series UHSAAs

**Figure 2:**
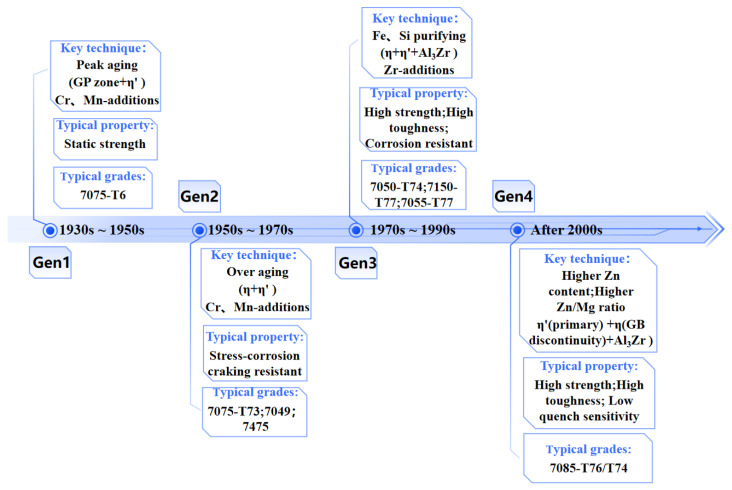
The developmental history of the 7xxx series UHSAAs and their corresponding representative aircraft applications.

Al–Zn–Mg alloys first attracted attention in the early 1930s; however, their application was initially limited by a high susceptibility to stress corrosion cracking (SCC) [32]. Subsequently, Webber introduced trace amounts of Cu into the Al–Zn–Mg system, which markedly improved corrosion resistance while retaining high tensile strength (up to 588 MPa) [33]. Further alloying with elements such as Cr and Mn led to the development of new UHSAAs by refining grain structures and improving microstructural homogeneity. As a result, Al–Zn–Mg–Cu alloys became a cornerstone material for aerospace applications [34]. Figure 2 illustrates the evolution of 7xxx series UHSAAs, with the chemical compositions of several representative alloys presented in Table 1. In the 1950s, the United States developed the 7075 alloy for aircraft engines and vertical tail structures, marking the emergence of the first-generation commercial Al–Zn–Mg–Cu alloys. The successful application of 7075-T6 in the B-29 bomber represented a milestone in the development of 7xxx series Al alloys [35,36]. In 1969, Aluminum Company of America (Alcoa) developed the second-generation 7475 alloy by significantly reducing Si and Fe impurity levels in the 7075 system [35,36]. Owing to its high-purity design and over-aging treatments, 7475 exhibited markedly improved fracture toughness and resistance to SCC, becoming the toughest 7xxx alloy of its time and finding widespread application in wing skins and spars of F-16 fighters [35,36].

However, the presence of Cr and Mn in 7475 alloy resulted in high quench sensitivity. To further optimize comprehensive mechanical performance, Alcoa introduced the third-generation 7050 alloy in 1971 by replacing Cr with Zr for grain refinement while increasing the Cu content and the Zn/Mg ratio. This alloy was extensively applied in load-bearing structures of aircraft such as the Boeing 777 and F/A-18 [3,8,38]. In parallel, the 7150 alloy was developed by further reducing Fe and Si impurities while maintaining Zn, Mg, and Cu contents near the upper limits of the 7075 specification, thereby achieving a balanced combination of high strength and corrosion resistance [38]. In the 21st century, Alcoa introduced the fourth-generation ultra-high-strength 7085 alloy. Characterized by a high Zn content and reduced Cu and Mg, 7085 exhibits significantly lower quench sensitivity and improved uniformity across thick sections, making it well suited for large integral aircraft structures and a continuing focus of research [39,40].

### 2.2. Al-Li Series UHSAAs

**Figure 3:**
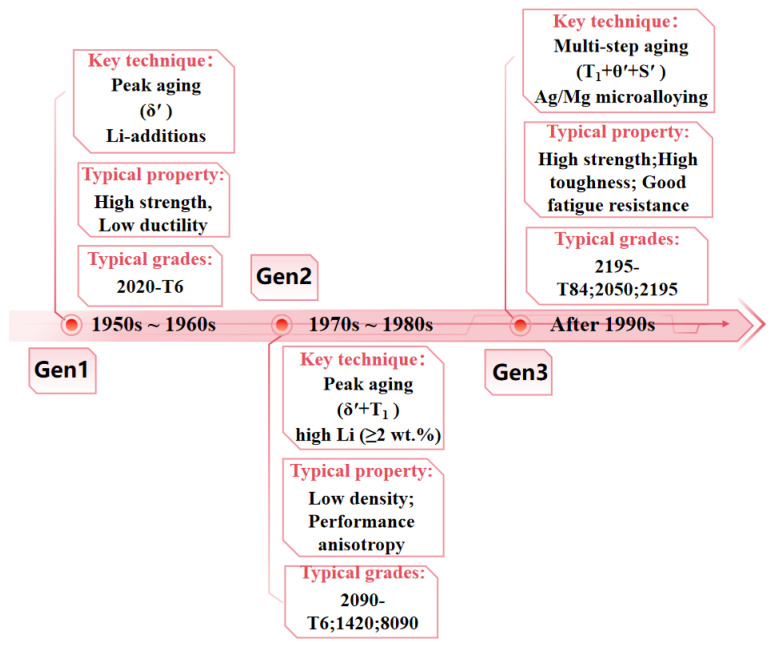
The developmental history of the Al–Li series UHSAAs and their corresponding representative aircraft applications.

The development of Al–Li alloys dates back to 1924, marking a century of evolution since the introduction of the first Al–Li alloy, Scleron (Al-12Zn-3Cu-0.6Mn-0.1Li). In 1955, Hardy and Silcock identified two primary strengthening phases in these alloys: δ′(Al_3_Li) and T_1_(Al_2_CuLi). This discovery provided a fundamental understanding of the precipitation hardening mechanisms, significantly accelerating research activities worldwide [41]. Figure 3 exhibits the evolution of Al–Li alloys. The first-generation Al–Li alloys, represented by the 2020 alloy (Al-4.5Cu-1.1Li-0.5Mn-0.2Cd), were developed by Alcoa in 1958 and applied to the wing and skin structures of the U.S. Navy RA-5C reconnaissance aircraft owing to their high strength and creep resistance. However, the formation of coarse impurity phases, such as Al_12_FeMn and Al_7_Cu_2_Fe, severely degraded ductility and fracture toughness, thereby limiting further engineering applications [30]. The development of second-generation Al–Li alloys was stimulated by the energy crisis of the 1970s, with the objective of replacing conventional Al alloys through weight reduction. These alloys were characterized by high Li (≥2 wt%) and low Cu (≤3 wt%) contents, resulting in an 8–10% reduction in density and a 10–16% increase in elastic modulus. Representative alloys include 1420, 2090, and 8090. Despite successful applications, such as 1420 alloy in MiG-29 fuselage structures and 8090 alloy in the fighter landing gear cabin door, the high Li content led to pronounced anisotropy and low toughness, which hindered widespread application [42].

Since the 1990s, third-generation Al–Li alloys have addressed these limitations through a “high-Cu, low-Li” design strategy. This is achieved by microalloying with trace elements (Ag, Zn, Ti, Zr, and Mn) and strictly controlling Fe and Si impurities. Typical alloys, including 2050, 2195, and 2198, have thus become the mainstream systems for advanced aerospace research and structural applications [43]. Table 2 presents the compositions, density, and registration date of typical third-generation Al–Li alloys. Compared with second-generation alloys, third-generation Al–Li alloys exhibit not only a superior strength–ductility balance but also reduced anisotropy and enhanced corrosion resistance. Notably, while Li addition enhances specific modulus and strength, it also increases the chemical reactivity of the alloy; even at limited concentrations (~1.0 wt.%), Li-bearing precipitates (such as the T_1_ phase) can act as initiation sites for localized corrosion, which is a critical factor for the long-term environmental durability of aerospace structures [11,23,44]. Third-generation Al–Li alloys are widely employed in critical structures of advanced spacecraft and military aircraft, including fuselage and wing skins, floor beams, stringers, flap ribs, and tail assemblies. For example, 2195 Al–Li alloy plates were used in the ultra-lightweight fuel tanks of the Space Shuttle Discovery, while 2196 Al–Li alloy forgings are applied in the main deck beams of the Airbus A380 and aircraft C919. Notably, replacing the conventional 2219 alloy with 2195 Al–Li for the Space Shuttle external tanks led to a substantial reduction in structural weight, thereby increasing payload capacity [44]. Table 3 compares the tensile properties of several Al–Li and 7xxx series UHHAAs.

### 2.3. Challenges in the Application of UHSAAs

A review of the development of UHSAAs reveals a consistent co-evolution between aerospace structural requirements and metallurgical advances. This synergy has not only driven progress in fundamental alloy research but has also significantly extended the service life of aerospace equipment. Compared with titanium alloys, UHSAAs offer ~20–30% lower density and significantly lower cost, enabling their substitution in certain aerospace structural components while maintaining yield strengths above 550 MPa. However, their relatively poor creep resistance above ~200 °C still limits applications in high-temperature environments [1,2,3,30]. With the increasing engineering application of 7xxx series alloys (e.g., 7085) and Al–Li alloys (e.g., 2050 and 2195), the strength of UHSAAs is approaching the theoretical limits of conventional precipitation-strengthening systems. Consequently, research efforts have shifted from the pursuit of maximum strength toward the synergistic optimization of strength, ductility, and damage tolerance [38].

Nevertheless, both 7xxx series alloys strengthened by η′/η precipitates and Al–Li alloys dominated by high-density T_1_ precipitates face common challenges, including limited ductility, low work-hardening capacity, and an increased tendency for strain localization with increasing strength. For highly alloyed 7085 alloys, the key challenge lies in the precise control of the size, distribution, and morphology of intragranular and grain-boundary precipitates (GBPs) to enhance YS while suppressing stress concentration and precipitation-free zones (PFZs) associated with high quench sensitivity [45]. Conversely, for Al–Li alloys such as 2050, the difficulty remains in mitigating strain localization caused by dislocation shearing of T_1_ and δ′ precipitates while addressing the pronounced mechanical anisotropy inherent in thick-section components [46,47].

Overall, while high precipitate density increases YS, it concurrently restricts dislocation mobility and uniform strain distribution; thus, the intrinsic strength–ductility trade-off remains a fundamental bottleneck. Moreover, in large integral components and complex service environments, both 7xxx series and Al–Li alloys exhibit pronounced microstructural and mechanical anisotropy. These challenges indicate that future development of UHSAAs must move beyond traditional single-mechanism strengthening strategies. By adopting multiscale microstructural design-incorporating microalloying, thermomechanical processing, and the coordinated regulation of precipitates and grain substructures, heterogeneous microstructures can be engineered to promote sustained work hardening [48]. Such strategies are expected to facilitate a breakthrough in the strength–ductility synergy and the overall in-service performance of new-generation UHSAAs.

## 3. Strategies for Regulating Microstructure and Mechanical Properties

### 3.1. Alloy Composition Optimization

The chemical composition of an alloy is the fundamental determinant of its microstructural evolution. For age-hardened Al–Li and 7xxx series aluminum alloys, precise control of the contents and ratios of alloying elements critically influences the type, volume fraction, and spatial distribution of strengthening precipitates. Specifically, microalloying additions do more than provide solid-solution strengthening via lattice distortion; they actively regulate precipitation kinetics, refine precipitate morphology, and optimize inter-precipitate spacing during aging, thereby promoting a favorable strength–ductility synergy. Consequently, chemical composition design guided by precipitation behavior represents one of the effective strategies for the mechanical performance optimization of UHSAAs.

In Al–Zn–Mg–Cu alloys [49], the primary alloying elements Zn and Mg are key constituents of the strengthening precipitates η′ (MgZn_2_, typically formed when the Zn/Mg mass ratio ≥ 2.2) and T′ (Al_2_Zn_3_Mg_3_, usually formed when Zn/Mg < 2.2). The individual addition of Zn or Mg has a limited effect on overall mechanical performance, whereas their combined addition at an appropriate ratio (Zn/Mg ≈ 2.5–2.7) effectively refines strengthening precipitates and markedly enhances alloy strength. However, when the total Zn + Mg content exceeds ~10 wt%, fracture toughness and corrosion resistance tend to deteriorate. These observations indicate that the Zn/Mg ratio plays a critical role in balancing strength and toughness in Al–Zn–Mg–Cu alloys [50,51]. Li and Engdahl [52,53] reported that, within a certain range, increasing the Zn/Mg ratio results in finer precipitates with higher number density, thereby improving mechanical properties. Zou et al. [54] further demonstrated that the Zn/Mg ratio alters the precipitation sequence in Al–Zn–Mg–Cu alloys and that precise control of this ratio to optimize the balance between η′ and T′ precipitates is an effective strategy for achieving high strength–ductility synergy. In their work [54], T′ precipitates dominate at low Zn/Mg ratios (<1.5), whereas η′ becomes the primary strengthening phase at high Zn/Mg ratios (>4.4). Notably, at a Zn/Mg ratio of 2.86, η′ and T′ precipitates coexist with the highest number density and the narrowest PFZ, resulting in a maximum tensile strength of 609 MPa and an EL of 6%.

**Figure 4:**
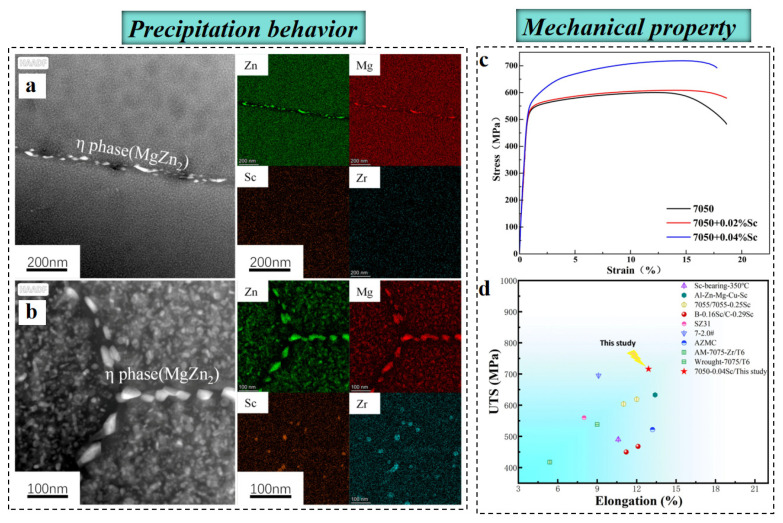
Effect of trace Sc addition on precipitation behavior (**a**,**b**) and mechanical property (**c**,**d**) of Al-6Zn-2Mg-2Cu-0.1Zr alloy, as well as the tensile strength and elongation of the 7050-0.04Sc alloy in comparison with those of other reported 7xxx-series Al alloys: (**a**) 7050 alloy; (**b**) 7050-0.04Sc alloy [59].

Moreover, microalloying 7xxx series alloys with trace Ti, Zr, Cr, Mn, Ag, and rare earth elements (Sc, Er, Y) achieves concurrent grain refinement, recrystallization suppression and precise tailoring of aging precipitates, thus synergistically optimizing grain structure and precipitation behavior and yielding balanced strength and ductility [55,56,57,58,59,60]. As shown in Figure 4, Huang et al. [59] demonstrated that trace addition of Sc (0.04 wt%) to the Al-6Zn-2Mg-2Cu-0.1Zr alloy facilitates the formation of Al_3_(Sc, Zr) particles during homogenization, which effectively impede both dislocation motion and grain boundary migration. As a result, the tensile strength and YS of the Al–Zn–Mg–Cu–Zr–Sc alloy increase by 20.9% and 24.3%, respectively, attaining values of 716 MPa and 640 MPa. Jiang et al. [58] reported that the addition of the rare earth element Y to a 7055 alloy induces the formation of a dual-phase microstructure composed of Al_8_Cu_4_Y and Al_3_(Y, Zr). During deformation, the Al_8_Cu_4_Y phase facilitates dynamic recrystallization by acting as nucleation sites, whereas the Al_3_(Y, Zr) phase pins subgrain boundaries and inhibits recrystallized grain growth. The synergistic action of these phases leads to the formation of a heterogeneous lamellar microstructure, enabling an excellent combination of high strength (695 MPa) and high ductility (16.6%). This outstanding mechanical response originates from hetero-deformation-induced hardening (HDI) associated with strain partitioning among regions with distinct microstructural characteristics [61,62]. Accordingly, the concept of hierarchical microstructural heterogeneity was proposed, in which strain delocalization is maximized through HDI, thereby overcoming the intrinsic strength–ductility trade-off.

**Figure 5:**
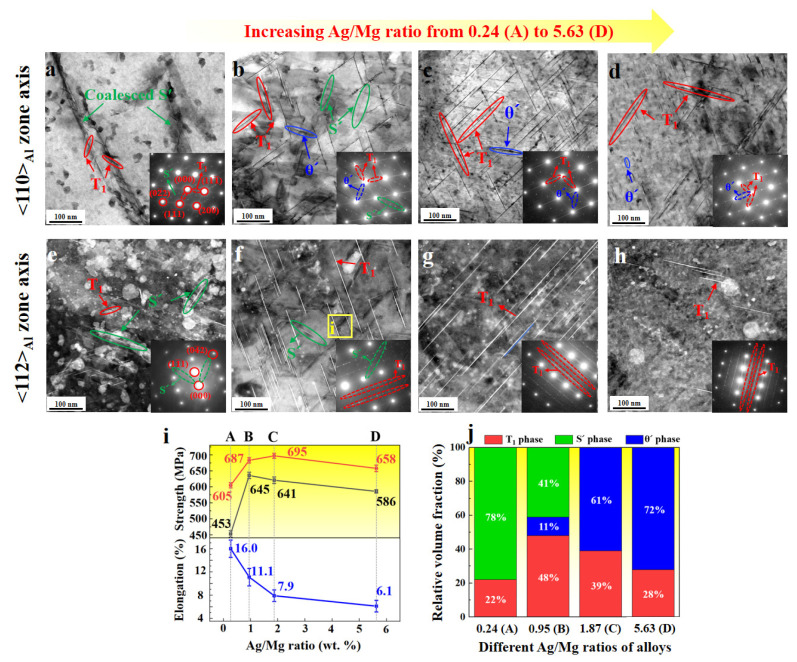
STEM-BF images viewed along [110]_Al_ (**a**–**d**) and [112]_Al_ (**e**–**h**) of samples with different Ag/Mg ratios, mechanical properties of alloys A–D. (**i**) and their relative volume fraction of precipitates (**j**). Specific Ag/Mg ratios are (**a**,**e**) 0.24, (**b**,**f**) 0.95, (**c**,**g**) 1.87, and (**d**,**h**) 5.63 [64].

Compared with 7xxx series UHSAAs with η′ and T′ as the dominant precipitates, Al–Li alloys exhibit a more complex variety of precipitates owing to Li addition and multi-element microalloying with Mg, Ag, Zr, Mn, and Zn [63,64,65,66,67,68,69]. Beyond the Li-based precipitates such as T_1_, δ′ (Al_3_Li), and δ (AlLi) phases, they also consist of various other phase structures, including θ′ (Al_2_Cu), GP zones, S′ (Al_2_CuMg), β′ (Al_3_Zr) phases, and Al_3_(Zr, Sc) composite phases. Our previous work provided new insights into improving the mechanical properties of Al–Cu–Li alloys by optimizing the Ag/Mg mass ratio (Figure 5) [64,65]. It was shown that variations in the Ag/Mg ratio can modify the precipitation sequence. Notably, at an Ag/Mg ratio of 0.95, T_1_, S′, and θ′ precipitates are uniformly dispersed within the matrix (Figure 5b,f). This multimodal precipitation effectively regulates dislocation motion and enhances ductility [64]. Compared with alloys strengthened by a single T_1_ or θ′ phase, the concurrent and homogeneous distribution of these three precipitate types enables an excellent combination of high strength (YS = 645 MPa) and good ductility (EL = 11.1%) (Figure 5i).

Zeng et al. [46] demonstrated that Mn microalloying effectively regulates recrystallization behavior and texture evolution in rolled Al–Cu–Li alloys, thereby enhancing strength–ductility synergy. Mn additions promote the precipitation of Al–Cu–Mn dispersoids during homogenization and subsequent thermomechanical processing, which act as effective heterogeneous nucleation sites for recrystallization during solution treatment [70]. As a result, the average recrystallized grain size is reduced by approximately 21%, accompanied by a significant suppression of mechanical anisotropy in the as-rolled condition. Notably, at an optimal Mn content of 0.4 wt%, the alloy exhibits the lowest anisotropy and good mechanical performance, with YS and EL reaching 551 MPa and 12.0% in the rolling direction and 528 MPa and 12.7% in the transverse direction, respectively.

To further enhance the efficiency and reliability of alloy development, modern alloy design increasingly employs integrated computational–experimental strategies. Thermodynamic calculations based on the CALPHAD approach, together with kinetic and precipitation modeling, are widely employed to predict phase stability, solute partitioning, and precipitation sequences in complex Al–Zn–Mg–Cu and Al–Cu–Li systems. These tools enable efficient screening of alloy compositions and optimization of key alloying elements to promote desirable strengthening phases while suppressing coarse detrimental intermetallic compounds. Additionally, integrated computational materials engineering (ICME) frameworks and first-principles calculations provide deeper insights into solute interactions and precipitate stability. Recently, machine-learning-assisted alloy design has emerged as a promising approach to accelerate the discovery of new compositions by uncovering correlations among composition, processing parameters, and mechanical properties.

### 3.2. Novel Forming and Processing Strategies

**Figure 6:**
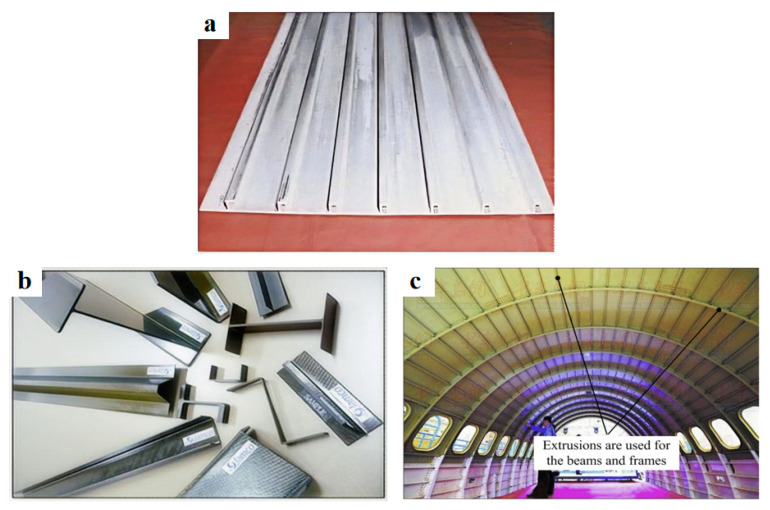
The applications of UHSLAAs [12]: (**a**) extruded 2090 aluminum alloy stiffened panels for the Space Shuttle’s external fuel tanks; (**b**) Al–Li alloy extruded profiles used as floor beams; (**c**) Al–Li alloy extruded profiles for C919 fuselage beams.

As critical lightweight structural materials in aerospace engineering, UHSAA components are primarily fabricated using conventional plastic forming techniques, including rolling, extrusion, and forging [71,72,73,74,75]. Figure 6 exhibits the the applications of UHSLAAs in the aerospace industry. These thermomechanical processes effectively eliminate casting defects (e.g., shrinkage cavities and porosity) while inducing work hardening and grain refinement to tailor the microstructure. However, with the growing demand for large-scale, integrated, complex, and high-precision aerospace structures, conventional forming methods increasingly suffer from limitations such as poor compositional and microstructural uniformity, pronounced texture-induced anisotropy, and a narrow processing window [73]. These drawbacks restrict their application in the fabrication of advanced aerospace components. To address these challenges, this review summarizes emerging plastic deformation techniques applicable to UHSAAs in aerospace applications.

**Figure 7:**
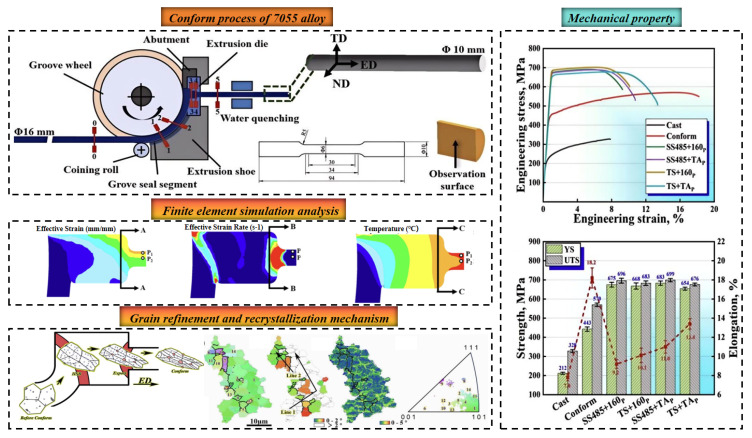
Microstructure evolution and mechanical property of 7055 alloy fabricated by continuous extrusion formation [81].

In recent years, severe plastic deformation (SPD) techniques, including equal channel angular pressing (ECAP) [76,77], high-pressure torsion (HPT) [78], and accumulative roll bonding (ARB) [79,80], have attracted considerable attention for their ability to fabricate bulk ultra-fine-grained (UFG) materials with tailored mechanical properties. A comparative summary of conventional and novel forming routes, as well as their corresponding mechanical properties, is presented in Table 4. Yang et al. [81] employed continuous extrusion forming to promote recrystallization in a 7055 aluminum alloy, achieving significant grain refinement, with the as-cast grain size reduced by 96.6% to ~3.4 μm, as shown in Figure 7. Subsequent solution treatment followed by two-step aging resulted in a synergistic enhancement of strength (YS = 683 MPa) and ductility (EL = 11.8%).

Compared with conventional SPD methods, repetitive continuous extrusion forming overcomes limitations related to billet size and processing efficiency, making it particularly suitable for Al–Zn–Mg–Cu alloys with inherently low hot workability [82,83]. Wen et al. [83] successfully applied repetitive continuous extrusion forming to a 7050 alloy and achieved simultaneous improvements in strength and ductility. Their results revealed that continuous dynamic recrystallization (CDRX) dominates grain refinement during extrusion, reducing the grain size from ~300 μm to ~40 μm after three passes. Notably, the combined effects of intense shear deformation and online quenching enable direct aging without a separate solution treatment, offering advantages in efficiency and cost. During direct aging, the introduction of high lattice distortion together with uniformly distributed precipitates endows the alloy with an excellent strength–ductility synergy.

**Figure 8:**
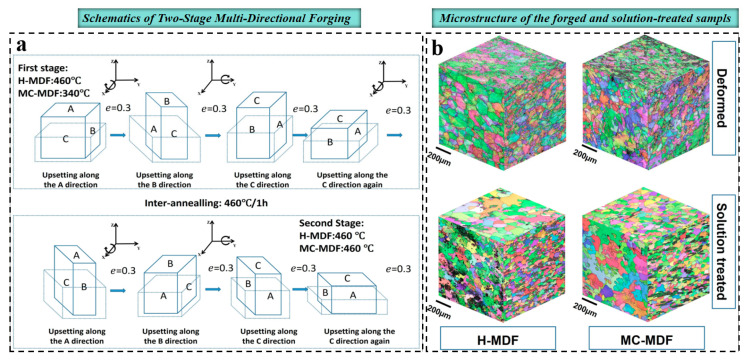
(**a**) Schematic of the MDF process; (**b**) microstructure of the 7085 alloy under different processing conditions [87].

Al–Li alloys are regarded as one of the most promising materials for transition rings in rocket propellant tanks. Currently, such components are predominantly manufactured by integral forging [84]. However, conventional forging routes (e.g., uniaxial compression) often result in insufficient deformation and highly non-uniform strain distribution in large ring billets, leading to deformation “dead zones”. These regions hinder the effective refinement of coarse grains and secondary particles, thereby degrading alloy ductility. Although SPD techniques, such as ECAP and HPT, can achieve substantial grain refinement, their application to large-scale engineering components such as ring billets remains limited by size and processing constraints. In contrast, multi-directional forging (MDF) [85,86,87], which involves successive upsetting and drawing steps along different directions, enables the introduction of high accumulated strain into large components and thus offers a practical pathway to realize SPD-like microstructural refinement. Generally, large-cross-Section 7085 alloy forgings exhibit over 40% anisotropy in mechanical properties, particularly in the vertical direction. Yin et al. [87] systematically investigated the effects of two-stage multi-directional forging (MDF) on the grain structure and mechanical properties of 7085 alloy, as shown in Figure 8. They found that isothermal medium-temperature composite MDF (MC-MDF) significantly improves the EL of 7085 forgings, especially along the height direction, and drastically reduces its anisotropy (IDAEL = 8.6%) in comparison with conventional isothermal hot MDF (H-MDF).

**Table 4:** Comparison of mechanical properties of different forming routes.

Alloy	Process Method	Heat Treatment State	YS (MPa)	UTS (MPa)	EL (%)
Al-7.8Zn-2.0Mg-2.4Cu [77]	Extrusion	T6	643	700	8.8
Al-4.7Cu-1.0Li-0.5Mg-1Zn [13]	Extrusion	T6	683	727	3.6
Al-3.3Cu-1.0Li-0.4Mg-0.3Ag [78]	Rolling	T8	470	519	15.5
7075 [80]	Rolling	T6	450	550	13
Al-7.8Zn-2.0Mg-2.4Cu [77]	ECAP	T6	664	730	8.5
7075 [80]	ARB	T6	603	652	7.1
Al-3.3Cu-1.0Li-0.4Mg-0.3Ag [78]	HPT	T8	700	711	<5%
7055 [81]	Continuous extrusion forming	T6	683	699	11.8
7050 [83]	Repetitive continuous extrusion	T6	419	486	14.9
7085 [87]	Multi-directional forging	T74	563	486	13.4

**Figure 9:**
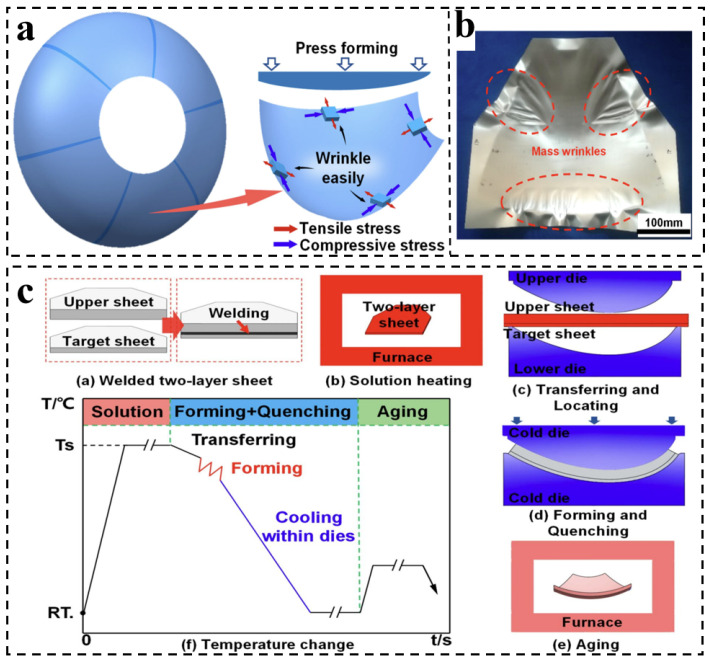
(**a**) Large-sized dome of rocket fuel tank [88]; (**b**) the occurrence of severe wrinkling and folding [88]; (**c**) schematic of the hot forming and quenching (HFQ) process [88].

Large-scale integral panels and skin components for aerospace vehicles require forming with complex curvatures, placing stringent demands on shape and dimensional accuracy. Particularly during the quenching of sheet components, non-uniform temperature fields and residual stress distributions often induce significant geometric distortion [88,89]. In response to this challenge, the hot forming and quenching (HFQ) process has been developed for the fabrication of complex components from heat-treatable high-strength Al alloys (Figure 9a), aiming to achieve both enhanced formability and high dimensional accuracy [90,91]. A schematic diagram of two-layer-sheet hot-forming-quenching integrated process is shown in Figure 9c. HFQ integrates hot forming with rapid in-die quenching, thereby suppressing distortion and springback while enabling an integrated “forming–strengthening” process. Zhang et al. [92] successfully fabricated 2A97 Al–Li alloy components using the HFQ process. In this route, the 2A97 alloy is first heated to the solution temperature and held to fully dissolve strengthening phases, producing a supersaturated solid solution. HFQ are then carried out simultaneously within the die under the combined action of applied pressure and cooling water, followed by artificial aging. Microstructural characterization revealed that the HFQ-processed 2A97-T8 alloy exhibits a dense distribution of fine, needle-like T_1_ precipitates, which markedly enhances its high-cycle fatigue (HCF) performance.

Nevertheless, a certain degree of springback has still been observed in large-scale rocket fuel tanks fabricated by HFQ, leading to reduced dimensional accuracy (Figure 9b). To overcome this limitation, Zheng et al. [93] proposed a quenching-forming and in-die creep aging (QICA) process derived from HFQ for the fabrication of large integral high-strength aluminum alloy panels. QICA combines hot stamping with subsequent in-die creep aging, significantly improving dimensional stability while retaining favorable mechanical properties. Compared with the HFQ process, QICA introduces a two-step in-die creep aging treatment after forming. By concurrently activating creep deformation and precipitation strengthening, springback is effectively suppressed and in situ hardening of the formed component is achieved.

### 3.3. Optimal Heat Treatment Process

With the continued advancement of aerospace systems, conventional heat treatment routes based on solution treatment followed by single-step aging are increasingly inadequate for achieving a balanced optimization of strength, ductility, and damage tolerance in UHSAAs. Consequently, advanced heat treatment and thermo-mechanical strategies emphasizing precise precipitation control and multiscale microstructural design have emerged as effective pathways to enhance their overall mechanical performance.

**Figure 10:**
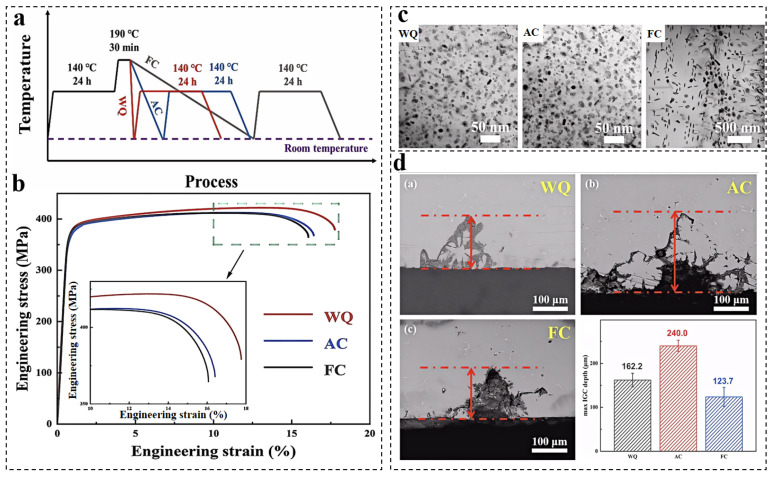
Schematic diagram (**a**), mechanical properties (**b**), precipitation behavior (**c**), and cross-sectional SEM images (**d**) of Al–Zn–Mg–Cu alloys under RRA treatment with different retrogression cooling modes [102]. The red dotted line in Figure 10d represents the mean maximum intergranular corrosion depth.

As age-hardening type UHSAAs, the aging process can control the morphology, size, and distribution of the precipitates. During the plastic deformation, the dislocation–precipitate interaction is a key factor determining its strength [94,95,96,97]. Moreover, the aging behavior also has a significant impact on the fracture toughness and SCC resistance. The aging treatments of 7xxx series Al alloys have progressively evolved from peak aging (T6) to over-aging conditions (T73/T76) and, more recently, to multistep schedules, aiming to balance strength and corrosion resistance [98]. Peak-aged T6 alloys derive their high strength from a dense distribution of fine, semi-coherent η′ precipitates within the matrix; however, the formation of continuous η precipitates along GBs significantly compromises SCC resistance [39]. Double-step aging promotes the coarsening and discontinuity of GBPs, thereby improving corrosion resistance at the expense of a typical 10–15% reduction in strength [99]. Retrogression and re-aging (RRA) treatment was subsequently developed to decouple matrix strengthening from grain-boundary precipitation control [100]. By introducing a short, high-temperature retrogression step between two T6 stages, RRA preserves the fine η′ precipitate population in the matrix while transforming GBPs into a coarser and more discontinuous morphology [101]. This tailored precipitation enables RRA-treated alloys to achieve corrosion resistance comparable to that of double-aged conditions while maintaining strength levels close to the T6. Zhang et al. [102] investigated the effects of retrogression cooling modes during RRA treatment on the microstructure, corrosion resistance, and mechanical properties of Al–Zn–Mg–Cu alloys. Figure 10 schematically illustrates the processing route, together with the associated microstructural evolution and property responses. Compared with air cooling (AC) and furnace cooling (FC), water quenching (WQ) was identified as a more favorable retrogression process, as it promotes the formation of finer and denser Tʹ/ηʹ precipitates [102].

For UHSAAs, moderate overaging during two-stage treatment (T7x) can improve HCF strength and the corrosion resistance of the alloy to some extent, but it is generally accompanied by a reduction in tensile properties [103]. This effect is particularly pronounced in alloys with a UTS exceeding 700 MPa, where the strength loss can reach ~8% [104]. To address this issue, Liu et al. [105] developed an interrupted aging heat treatment for an ultra-high-strength Al–Zn–Mg–Cu alloy. Compared with conventional T6 peak aging, this process involves a brief pre-aging step at a temperature close to the conventional peak-aging temperature, followed by a second aging stage at a lower temperature [106]. The results demonstrated that interrupted aging at 90 °C not only significantly enhances the HCF strength in T6 but also allows the alloy to achieve a UTS of 765 MPa while maintaining a tensile strength comparable to that of the T6 condition. Similarly, Zou et al. [107] reported that Al–Zn–Mg–Cu alloys subjected to interrupted aging exhibited an EL increase of approximately 68% relative to the conventional T6 while retaining a UTS comparable to the T6 state.

In contrast to 7xxx series alloys, whose heat-treatment strategies emphasize thermodynamic control of precipitate morphology—often compromising peak strength to improve corrosion resistance and damage tolerance via RRA treatments—the heat-treatment approach for Al–Cu–Li alloys is primarily driven by strain-induced heterogeneous nucleation, facilitating a uniform distribution of T_1_ precipitates [108]. Such a strategy, in combination with multiscale precipitate morphology regulation, effectively suppresses planar slip and contributes to the achievement of both ultra-high strength and ductility. To date, thermomechanical processing combining pre-deformation with artificial aging is widely employed to improve the strength–ductility synergy of Al–Li alloys [109,110,111,112,113]. Pre-deformation increases dislocation density, providing heterogeneous nucleation sites for T_1_ precipitation and suppressing GBP growth, thereby enhancing both strength and ductility. However, an excessive pre-deformation level has been shown to introduce grain-boundary stress concentrations due to dislocation migration and to cause heterogeneous precipitation under uniaxial stress [26,114,115], which significantly restricts ductility at strengths exceeding ~680 MPa [110,116].

**Figure 11:**
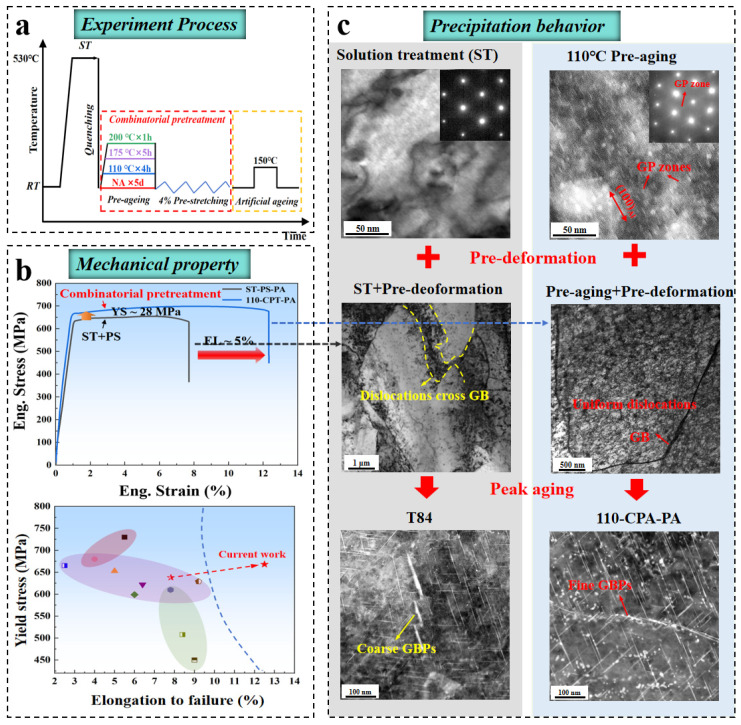
Heat treatment processes (**a**), corresponding mechanical properties (**b**), and precipitation behavior (**c**) of Al–Cu–Li alloys under conventional T8 and combinatorial pre-treatment [116].

A critical scientific question arises from these observations: Can the uniformity of the dislocation configuration introduced during pre-deformation be optimized through deliberate tailoring of the intragranular precipitate? Recent studies have demonstrated that low-temperature pre-aging at 100 °C facilitates the formation of GP I-type precursors in Al–Zn–Mg–Cu alloys [117]. These precursors act as effective heterogeneous nucleation sites for subsequent strengthening phases, thereby increasing YS by 184 MPa while simultaneously improving ductility by more than 10%. Similarly, natural aging prior to pre-deformation has been shown to suppress localized dislocation clustering in Al–Zn–Mg-Cu alloys by promoting GP zone formation, resulting in a finely dispersed and more uniform precipitate distribution [26]. However, in Al–Mg–Si alloys, natural aging tends to promote solute clustering that depletes the solute atoms required for subsequent precipitation, invariably leading to a reduction in strength. By contrast, short-term pre-aging immediately after quenching can effectively mitigate this detrimental effect [118]. Motivated by these insights, a series of coupled pre-aging and pre-deformation strategies were therefore proposed for T8-treated Al–Li alloys, with particular emphasis on the role of pre-aging in regulating T_1_ precipitation behavior and its interaction with dislocations, as schematically shown in Figure 11a. The results indicate that high-temperature pre-aging (175–200 °C) induces a high density of T_1_ precipitates, which leads to pronounced dislocation entanglement during pre-deformation and subsequently accelerates T_1_ coarsening during artificial aging. In contrast, low-temperature pre-aging at 110 °C promotes the formation of GP zones that accommodate plastic strain predominantly via a dislocation-shearing mechanism [116]. This mechanism results in a more homogeneous dislocation configuration, thereby creating favorable conditions for the uniform nucleation and growth of precipitates during subsequent aging (Figure 11c). Compared to the conventional T8 process, this optimized strategy, combining 110 °C pre-aging with 4% pre-deformation, enhances YS and EL by 4.4% and 60.3%, respectively (Figure 11b).

As mentioned above, in Al–Cu–Li alloys, pre-aging at 110 °C prior to pre-deformation promotes a relatively uniform dislocation configuration through interactions between metastable GP zones and dislocations. This finding suggests that the precipitation of nanoscale, shearable precipitates, characterized by a high aspect ratio and dense spatial distribution within the matrix, represents an effective strategy for enhancing both the strength and ductility of Al–Li alloys [119,120]. However, metastable GP zones partially dissolve during pre-deformation and rapidly transform into θ′ precipitates during subsequent artificial aging [121], leading to precipitates dominated by shear-resistant T_1_ and θ′ phases and a consequent loss of ductility.

**Figure 12:**
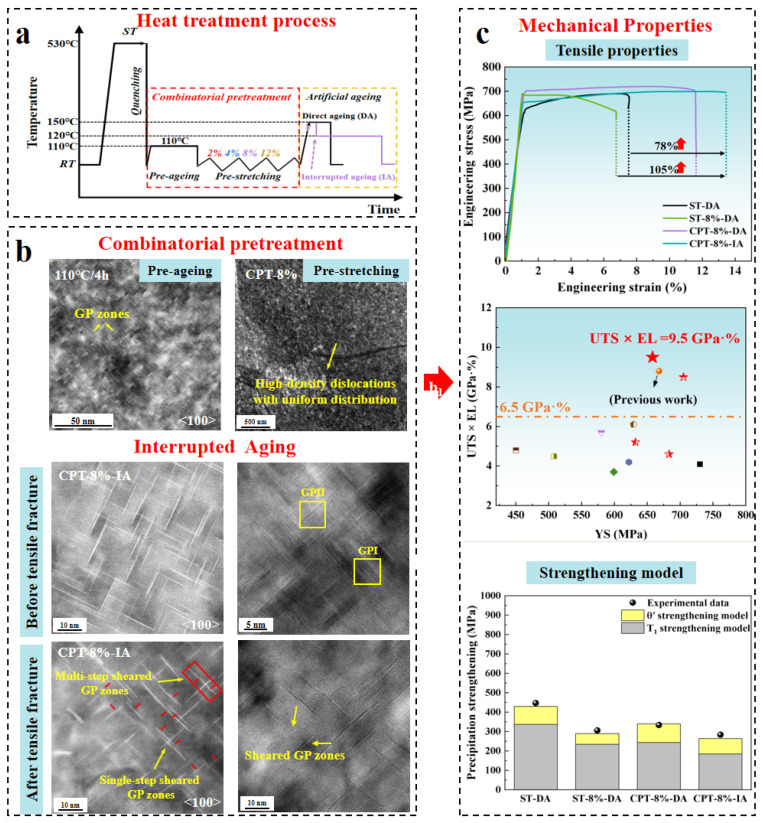
Heat treatment processes (**a**), precipitation behavior (**b**), and corresponding mechanical properties (**c**) of Al–Cu–Li alloys under a novel thermomechanical treatment [122].

To overcome this limitation, a cooperative strengthening strategy combining 110 °C pre-aging, controlled pre-deformation (2–12%), and low-temperature interrupted aging was developed (Figure 12a) [122]. The optimized heat treatment process (110 °C pre-aging + 8% pre-deformation + 150 °C/20 min + 120 °C/64 h) effectively alleviates local stress concentration associated with dislocation entanglement, resulting in a high-density and homogeneous dislocation configuration. Subsequent interrupted aging enables secondary GP zone precipitation, producing a multiscale heterogeneous microstructure consisting of GP zones (~9 nm) and T_1_ precipitates (~39 nm) (Figure 12b). During plastic deformation, GP zones–dislocations interactions lead to the strain-induced dissolution of approximately 45% of the GP zones, with the released Cu atoms re-dissolving into the Al matrix. This “weak pinning–multiple shearing–re-solution” mechanism not only extends the average dislocation mean free path but also enhances the intrinsic plasticity of the Al matrix [121], thereby leading to a pronounced improvement in EL. As a result, the alloy achieves a YS of 657 MPa, a UTS of 700 MPa, and an EL of 13.5%, corresponding to nearly a twofold increase in ductility compared with the conventional 2050 alloy while maintaining a comparable strength level (Figure 12c).

## 4. Mechanism of Strength–Ductility Synergy

The primary strengthening mechanisms in UHSAAs include grain boundary strengthening, solid-solution strengthening, deformation strengthening, and precipitation strengthening [123,124,125]. All of these mechanisms significantly influence alloy ductility and generally involve an inherent strength–ductility trade-off. Achieving an optimal strength–ductility synergy relies on precipitation strengthening as the dominant mechanism. Precise control of finely dispersed nanoscale precipitates enables high strength while suppressing strain localization, assisted by moderate grain boundary strengthening and rational contributions from solid-solution strengthening and deformation strengthening. The strength–ductility synergy of UHSAAs can be enhanced through the following three strategies.

### 4.1. Regulation of Shearable Precipitates

**Figure 13:**
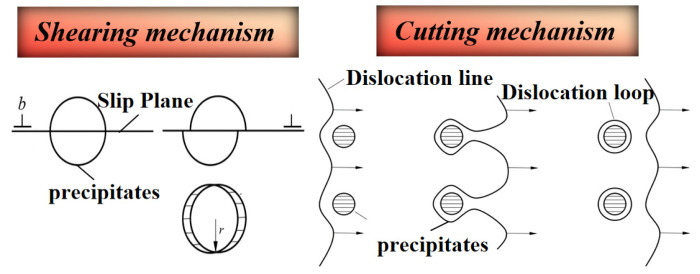
Schematic of dislocation–precipitate interaction mechanism.

Aging treatments promote the formation of a high density of finely dispersed precipitates, resulting in pronounced precipitation strengthening by impeding dislocation motion. Dislocation–precipitate interactions are commonly classified into two mechanisms: dislocation shearing and dislocation bypassing (Figure 13). In Al–Cu–Li alloys, coherent δ′, GP zones, and fine semi-coherent T_1_ precipitates can be sheared by gliding dislocations, enabling cooperative deformation with the matrix. This process increases the precipitate–matrix interfacial energy and introduces additional energy barriers for dislocation motion, thereby enhancing strength [126]. Single-layer T_1_ precipitates and GP zones can be sheared by dislocations [127]. With continued aging, T_1_ precipitates grow to a critical size, leading to a substantial increase in their shearing resistance. Dislocations then undergo pronounced bending and local stress accumulation, rendering the shearing mechanism energetically unfavorable. Consequently, dislocations bypass the precipitates by forming Orowan loops [128]. Semi-coherent θ′ precipitates are generally resistant to shearing, and dislocation–θ′ interactions therefore predominantly occur via the bypass mechanism. Yang et al. [119] reported that bypassing of coarse θ′ precipitates induces local lattice rotations of ~2–5° and severe lattice distortions. Moreover, dislocation pile-up at θ′/Al interfaces promotes void nucleation and growth, ultimately degrading alloy ductility [119].

Based on the tensile ductility model proposed by Chan et al. [129] and Liu et al. [130], brittle inclusion phases in Al alloys are prone to fracture during plastic deformation. Consequently, these brittle phases act as crack initiation sites, thereby directly influencing the fracture strain (*ε*_f_). This can be expressed by Equation (1):

(1) $$\varepsilon_{f}={\frac{1}{{\tilde{\varepsilon }}_{e}\left(\theta \right)}\left[\frac{I}{0.405\pi h}\right]}^{\frac{1}{n+1}}{\left[\frac{\lambda_{c}}{2r_{c}}\;-\;1\right]}^{\frac{1}{n+1}}\frac{\tilde{\varepsilon }}{2}$$

where $\lambda_{c}$ and $r_{c}$ denote the inter-particle spacing and size of the inclusions, respectively, and ${\tilde{\varepsilon }}_{e}$ is the effective value of a normalized parameter. The terms *I* and *h* are functions of the strain hardening exponent *n*, $\tilde{\varepsilon }$ denotes the critical local plastic strain required for material fracture induced by microcracks.

In Al–Li alloys, the deformation incompatibility between the matrix and the various types of precipitates or secondary phases induces geometrically necessary dislocations (GNDs) at the phase interfaces, generating local plastic strain. Fracture of the aluminum matrix occurs once the GND density reaches a critical value [130]. The relationship between $\tilde{\varepsilon }$ and the critical GND density $\rho_{g}^{c}$ is given by Equation (2) [131]:

(2) $$\tilde{\varepsilon }\;=\;0.25b\lambda_{p}\rho_{g}^{c}$$

where $\lambda_{p}$ represents the inter-precipitate spacing and b is the Burgers vector. According to Equation (2), for a given critical dislocation density, the local critical strain is primarily governed by precipitate size. For T_1_ precipitates with a {111}_a_ habit plane in Al–Li alloys, the inter-particle spacing λ is given by Equation (3) [132]:

(3) $$\lambda =\sqrt{\pi \mathrm{dt}/3\sin70.5{}^{\circ}f_{v}}$$

where *d*, *t*, and *f_v_* are the diameter, thickness, and volume fraction of the T_1_ phase, respectively. For the non-shearable θ′ phase precipitating on {100}_a_ planes, the spacing is expressed as Equation (4) [133]:

(4) $$\lambda \;=\;\frac{1.030}{\sqrt{\mathrm{Nd}}}\;-\;\frac{\pi d}{8}\;-\;1.061t$$

where *N* is the number density of the θ′ phase. From the aforementioned equations, it is evident that for non-shearable θ′ precipitates, any microstructural evolution leading to a reduction in inter-particle spacing λ (e.g., increasing the number density, diameter, or thickness) decreases the critical local plastic strain, thereby reducing overall ductility. In contrast, for shearable T_1_ precipitates, although an increase in volume fraction *f_v_* reduces the local plastic strain, increasing their diameter and thickness conversely contributes to a higher critical local plastic strain, thus improving plasticity. Therefore, while dislocations struggle to accumulate around small, shearable T_1_ precipitates, an appropriate increase in T_1_ diameters may enhance ductility.

On the other hand, the strong interaction between non-shearable precipitates and dislocations leads to the accumulation of Orowan loops and increased local stress. This results in stress/strain concentration zones that readily inducing microvoid formation, consequently degrading ductility and fracture toughness [119,127]. Conversely, for shearable metastable GP zones and fine T_1_ phases, the local shear stress is significantly lower than that around θ′ phases. Although this reduces the obstacle strength against dislocation motion, it effectively alleviates local dislocation pile-ups, thereby improving ductility. This mechanism explains why under-aged Al–Li alloys typically exhibit superior ductility, albeit with lower strength, compared to their peak-aged counterparts. Therefore, tailoring the morphology and distribution of the T_1_ precipitates—specifically promoting the precipitation of shearable T_1_ plates with high aspect ratios—serves as an effective strategy for enhancing the strength–ductility synergy of Al–Li alloys.

### 4.2. Regulation of Solute Atom Cluster

The aging precipitation kinetics of UHSAAs generally follow a continuous sequence of supersaturated solid solution → solute atom clustering → precipitate nucleation and growth. Research indicates that the strengthening effect is minimal when solutes exist as individual atoms within the solid solution. Conversely, while the formation of nanoscale phases with a complete crystal structure can significantly enhance strength, it often leads to a reduction in ductility [134,135]. Intermediate solute clusters (i.e., metastable GP zones) exhibit a unique capacity for coordinating strength and ductility [136,137].

The addition of Mg induces a pronounced cluster-strengthening effect during natural aging, which is commonly attributed to the formation of Mg–Cu clusters that progressively evolve into Cu-rich metastable GP zones; accordingly, Mg–Cu clusters are widely regarded as precursors to GP zones [136]. Sun et al. [138] introduced a high concentration of vacancies into 2024 alloy via cyclic deformation at room temperature, inducing the dynamic precipitation of high-density, ultrafine (1–2 nm) solute clusters. Without conventional aging, the alloy achieved tensile strength comparable to that of the T6 state while maintaining EL similar to the as-quenched state, thereby achieving an excellent strength–ductility synergy. Consistent observations have been reported in other alloy systems. Yang et al. [139] found in Al–Sc alloys that although the strengthening potency of individual solute atoms is far lower than that of precipitates, the number density of solute clusters is an order of magnitude higher than that of precipitates. This significantly enhances alloy strength while effectively mitigating the detriment to ductility. Furthermore, Zhang et al. [140] observed that prolonged natural aging (~1440 h) led to the formation of a high density of Zn–Mg clusters (3.13 × 10^24^ m^−3^), significantly improving strain capacity and ductility. Pogatscher et al. [141] demonstrated in Al–Mg–Si alloys that quenched-in vacancies promote GP zone formation during early aging, which subsequently facilitates a more favorable strength–ductility balance during transformation to the β″ phase.

### 4.3. Regulation of Grain Boundary Precipitation Behavior

**Figure 14:**
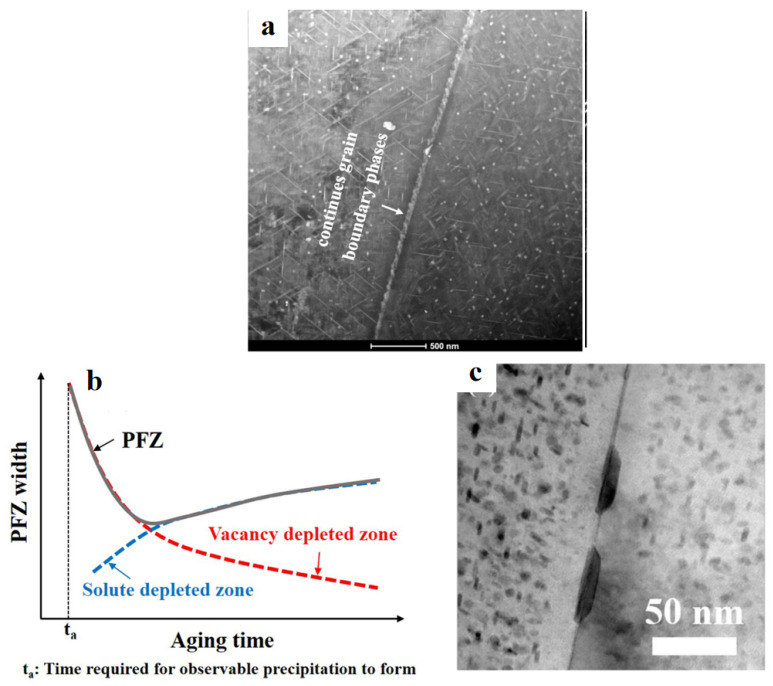
Precipitation behavior at GBs: (**a**) precipitation morphology at subgrain boundaries [142]; (**b**) schematic representation of PFZ formation; (**c**) PFZ in Al–Zn–Mg–Cu alloy [102].

The morphology and distribution of GBPs, along with the width of PFZs, exert a significant influence on the ductility of UHSAAs. Along subgrain boundaries in Al–Li alloys, T_1_ precipitates with a small inclination to the boundary plane are commonly observed. When viewed along the [110]_α_ direction, the angle between two T_1_ variants in the matrix is 70.5°, whereas T_1_ precipitates nucleated at subgrain boundaries typically form a much smaller angle with the boundary, on the order of ~21–25° (Figure 14a) [142]. In contrast to precipitation at subgrain boundaries, GBP is prone to inducing the PFZ formation, which plays a critical role in determining the ductility of UHSAAs. The formation mechanisms of PFZs primarily include the solute depletion mechanism and the vacancy depletion mechanism [143]. The solute depletion model postulates that the continuous consumption of alloying elements by coarsening GBPs leads to local solute exhaustion in the vicinity of GBs, thereby forming PFZs. Conversely, the vacancy depletion model emphasizes the essential role of vacancies in precipitate nucleation, as vacancies serve both as heterogeneous nucleation sites and as diffusion pathways for solute atoms. During quenching, GBs act as effective vacancy sinks, resulting in vacancy depletion near the boundaries and suppressing the nucleation of metastable precipitates, thereby giving rise to PFZs.

In UHSAAs, PFZ formation is generally governed by the combined action of these two mechanisms (Figure 14b). Under conditions of rapid quenching and short aging times, vacancy depletion dominates PFZ formation; with decreasing quench rate and prolonged aging, enhanced solute diffusion renders solute depletion increasingly prevalent (Figure 14c) [102]. The presence of PFZs generally leads to a pronounced deterioration in ductility. Due to their intrinsically low strength, PFZs provide limited resistance to dislocation motion and crack initiation. During deformation, dislocations readily accumulate near GBs, and the resulting pile-ups generate stress concentrations at secondary particles, thereby promoting crack initiation. Cracks preferentially propagate along PFZs, leading to intergranular fracture and, consequently, a reduction in ductility [144,145].

## 5. Future Prospects

Next-generation UHSAAs for aerospace applications are moving toward synergistic performance optimization and cost-effective industrialization. The development paradigm has shifted from pursuing strength alone to addressing lightweight, strength–ductility synergy, fatigue/damage tolerance, and environmental adaptability. To meet these rigorous demands, future research and engineering applications of 7xxx series and Al–Li alloys must embrace the following directions:

(1) Heterogeneous Microstructural Design: Transitioning from single-scale microstructure regulation to the deliberate construction of multiscale heterogeneous architectures, thereby exploiting the underlying mechanisms governing the simultaneous enhancement of strength, ductility, and damage tolerance [48].
(2) In-service Predictability: Systematic investigation of microstructural stability and mechanical property reduction under high-temperature exposure, corrosive environments, and cyclic loading, enabling alloy design to evolve from initial property optimization toward life-cycle service performance prediction [45,146].
(3) Process Integration: Integrating emerging processing technologies, such as additive manufacturing, severe plastic deformation, and powder metallurgy, to overcome the intrinsic performance and structural limitations associated with conventional manufacturing routes [73].
(4) Digital Intelligence: The adoption of digital intelligence through ICME frameworks that couple physics-based modeling with data-driven machine learning approaches is expected to enable precise performance tailoring and significantly accelerate the deployment of UHSAAs in next-generation aerospace systems [147,148,149]. Specifically, AI algorithms can predict complex precipitation kinetics—such as optimizing the Zn/Mg ratio in 7xxx alloys or managing the competition among T1, S′, and θ′ phases in Al–Li alloys—far faster than traditional trial-and-error methods. Machine learning models trained on robust datasets will identify optimal microalloying compositions and processing windows, bypassing exhaustive experimental iterations

Leveraging ICME frameworks that couple physics-based modeling with data-driven machine learning approaches should be used to achieve precise performance tailoring and accelerate the deployment of UHSAAs in next-generation aerospace systems [134,135,136].

## 6. Conclusions

This review summarizes recent advances in ultra-high-strength aluminum alloys for aerospace applications, with particular emphasis on the 7xxx series and Al–Li alloys. Driven by the increasing demand for lightweight and reliable aerospace structures, research efforts have gradually shifted from the pursuit of maximum strength toward achieving a synergistic balance of strength, ductility, and damage tolerance. Such synergy can be realized through three primary strategies: optimized alloy composition design, the adoption of advanced forming technologies, and the development of innovative thermomechanical treatments. Fundamentally, these improvements are governed by nanoscale microstructural regulation. Superior mechanical performance depends on the controlled distribution of shearable precipitates, the formation of solute atom clusters that promote uniform plastic deformation, and the optimization of grain boundary precipitation to suppress PFZs. Consequently, the transition from conventional processing routes toward multiscale heterogeneous microstructural design is expected to play a crucial role in the development of next-generation aerospace ultra-high-strength aluminum alloys.

## Figures and Tables

**Table 1:** Chemical compositions of typical 7xxx series UHSAAs [37].

Alloy	Mass Fraction (wt.%)							
	Zn	Mg	Cu	Cr	Mn	Ti	Zr	Al
7075	5.1–6.1	2.1–2.9	1.2–2.0	0.15–0.35	0.2–0.3	0.04	-	Bal.
7475	5.2–6.2	1.9–2.6	1.2–1.9	0.18–0.25	<0.06	<0.06	-	Bal.
7050	6.0–6.7	2.2–2.6	2.0–2.6	<0.01	<0.016	<0.06	0.12	Bal.
7150	5.9–6.2	2.3–2.4	2.0–2.2	-	0.01	0.03–0.05	0.12	Bal.
7055	8	2.2	2.2	-	-	-	0.12	Bal.
7085	7.0–8.0	1.2–1.8	1.3–2.0	0.04	0.04	0.06	0.08–0.15	Bal.

**Table 2:** Compositions, density, and registration date of typical 3rd generation Al-Li alloys [12,44].

Alloy	Mass Fraction (wt.%)								ρ g/cm^3^	Date Year
	Li	Cu	Mg	Ag	Zn	Zr	Mn	Other		
2195	1.0	4.0	0.4	0.4	-	0.11	-	Ti0.1	2.70	1992
2297	1.4	2.8	≤0.25	-	≤0.5	0.11	0.3	-	2.65	1997
2196	1.75	2.9	0.5	0.4	≤0.35	0.11	≤0.35	-	2.63	2000
2098	1.05	3.5	0.53	0.43	0.35	0.11	≤0.35	-	2.70	2000
2050	1.0	3.6	0.4	0.4	≤0.25	0.11	0.35	-	2.70	2004
2198	1.0	3.2	0.5	0.4	≤0.35	0.11	≤0.5	Ti0.1	2.69	2005
2296	1.6	2.45	0.6	0.43	≤0.25	0.11	0.28	-	2.63	2010
2055	1.15	3.7	0.4	0.4	0.5	0.11	0.3	-	2.70	2011
2060	0.75	3.95	0.85	0.25	0.4	0.11	0.3	-	2.72	2011
2076	1.5	2.35	0.5	0.28	≤0.3	0.11	0.33	-	2.64	2012
2A97	1.5	3.5	0.4	-	0.5	0.12	0.3	-	/	/

**Table 3:** Mechanical properties of 3rd Al–Li and 7xxx series UHHAAs [8,38].

Alloy	Forming Process	Heat Treatment	Mechanical Property		
			*R_p_*_0.2_ (MPa)	*R*_m_ (MPa)	A (%)
7055	Plate (L)	T7751	634	648	11.0
7075	Plate (TL)	T651	505	570	11.0
7050	Rod	T8	655	689	7.5
7150	Plate (L)	T7751	634	675	12.0
	Plate (LT)	T76511	606	606	11.0
2050	Sheet (L)	T8	507	552	10.3
	Rod	T8	666	687	6.3
2055	Sheet (L)	T8	543	582	7.6
	Rod	T8	665	675	2.5
2195	Rod	T6	570	663	9.6
	Rod	T8	674	683	6.6
2095	Sheet (L)	T8	660	700	8.0
	Rod	T6	683	718	3.8

## Data Availability

No new data were created or analyzed in this study. Data sharing is not applicable to this article.
